# Peripheral Blood Mononuclear Cell Expression Profile of Proliferation-, Ferroptosis-, and Immune-Related Targets in Breast Cancer

**DOI:** 10.3390/medicina62101831

**Published:** 2026-09-23

**Authors:** Rabia Demirtaş, Murat Kızılkaya, Eda Balkan, Mahmut Aktürk, Mert Yüksel, Abdulkadir Aktepe, Murat Kartal, Hasan Türkez

**Affiliations:** 1 Department of Pathology, Faculty of Medicine, Atatürk University, 25240 Erzurum, Türkiye; rabia.demirtas@atauni.edu.tr; 2 Department of Medical Biology, Ağrı İbrahim Çeçen University, 04000 Ağrı, Türkiye; mkizilkaya@agri.edu.tr; 3 Department of Medical Biology, Faculty of Medicine, Atatürk University, 25240 Erzurum, Türkiye; mahmutakturk25@hotmail.com (M.A.); hasanturkez@yahoo.com (H.T.); 4 Faculty of Medicine, Atatürk University, 25240 Erzurum, Türkiye; mertyuksel1907@gmail.com; 5 Department of General Surgery, Faculty of Medicine, Atatürk University, 25240 Erzurum, Türkiyem.kartal2587@hotmail.com (M.K.)

**Keywords:** breast cancer, peripheral blood mononuclear cells, gene expression profile, PTEN, ferroptosis, STAT3, miRNA, qRT-PCR

## Abstract

*Background and Objectives:* This study evaluated eight targets related to proliferation (miR-21, miR-210, PTEN, and mTOR), ferroptosis regulation (SLC7A11, GPX4, and miR-214), and immune regulation (STAT3) in bulk (unfractionated) peripheral blood mononuclear cells (PBMCs) from patients with breast cancer. Their exploratory discriminative performance and associations with clinicopathological characteristics, including immunohistochemical parameters, were also examined. *Materials and Methods:* Target expression was quantified by qRT-PCR in PBMCs from 41 patients with histopathologically confirmed breast cancer and 30 healthy controls. Immunohistochemistry-defined surrogate subtypes (hereafter, IHC-defined subtypes) were assigned according to ER, PR, HER2 and Ki-67 status. Between-group comparisons were performed using the independent-samples *t*-test or the Mann–Whitney U test; multiple comparisons were corrected using the Benjamini–Hochberg false discovery rate (BH-FDR) method. Exploratory discriminative performance was determined by ROC analysis with 2000-iteration bootstrap confidence intervals. Directional consistency was examined in two independent PBMC GEO datasets (GSE27562, GSE47862); this was a directional comparison rather than formal external validation. *Results:* Seven of the eight targets differed significantly after BH-FDR correction: STAT3, mTOR, SLC7A11, miR-21, and miR-210 were upregulated, whereas PTEN and miR-214 were downregulated. In this exploratory, single-cohort case-control study, the largest exploratory single-marker AUCs were observed for STAT3, PTEN, miR-210 and SLC7A11. No association with clinicopathological parameters survived multiple-testing correction. In a post hoc exploratory comparison, PBMC PTEN levels were lower in TNBC (*n* = 13) than in non-TNBC cases (*n* = 28), but this difference did not remain significant after BH-FDR correction (Mann–Whitney U, *p* = 0.024; BH-FDR *p* = 0.193). *Conclusions:* Breast cancer was associated with distinct expression patterns in bulk PBMCs, including increased miR-21 and mTOR expression with decreased PTEN expression, increased SLC7A11 with decreased miR-214 expression, and increased STAT3 expression. Five significant pairwise correlations were identified among the measured targets. Because these findings were derived from transcript-level measurements in unfractionated PBMCs, they do not establish functional pathway activation or ferroptosis resistance. Lower PBMC PTEN expression in TNBC reached nominal but not FDR-corrected significance, so no clinicopathological association is claimed.

## 1. Introduction

Breast cancer is the most frequently diagnosed cancer in women worldwide and one of the leading causes of cancer-related death; according to 2022 estimates, it accounts for approximately 11.6% of all cancers in women [1]. Although early diagnosis is one of the most critical factors determining survival in breast cancer, current screening methods (such as mammography) have several limitations, including sensitivity, cost, and reduced performance in young women or those with dense breast tissue. This underscores the need for minimally invasive, repeatable biomarkers capable of reflecting systemic tumor biology.

It has been shown that the transcriptomic profile of peripheral blood mononuclear cells (PBMCs) changes markedly in the presence of breast cancer as a result of tumor–host interactions, and that this change reflects a systemic immune and metabolic response [2]. This observation suggests that expression patterns in peripheral blood may carry systemic information about tumor biology. Nevertheless, studies evaluating PBMC-based multi-gene expression panels in breast cancer—particularly for panels combining proliferation-, ferroptosis- and immune-related targets—are limited.

In this study, eight targets were selected from three areas of breast cancer biology that are frequently studied together. The first group comprises targets associated with proliferation and survival: the oncogenic microRNAs miR-21 and miR-210 are consistently upregulated in many malignancies, including breast cancer. miR-21 directly targets the tumor suppressor PTEN, leading to uncontrolled activation of the PI3K/AKT/mTOR survival pathway [3]; miR-210, regulated by hypoxia-inducible factor (HIF), has been defined as the principal “hypoxamir” that supports cell survival in the hypoxic tumor microenvironment [4]. The second group comprises targets associated with ferroptosis regulation: the cystine/glutamate antiporter SLC7A11 (xCT) feeds glutathione biosynthesis, thereby suppressing lipid peroxidation and ferroptosis; it is overexpressed in various cancers and confers resistance to cell death [5]. mTOR signaling has been described as an upstream regulatory component of this metabolic program.

The third group comprises targets associated with immune-metabolic regulation. STAT3 is a transcription factor that is activated via IL-6/JAK signaling in peripheral blood myeloid cells and governs the immunosuppressive program; it is closely associated with the expansion of myeloid-derived suppressor cells and immune evasion in the tumor microenvironment [6]. Because STAT3 has been implicated in both proliferation-associated and ferroptosis-associated processes in the literature, it was included as a target of particular interest. Our group has applied a peripheral blood non-coding RNA and inflammation-related gene profiling approach in other disease models, namely renal transplant rejection and multiple sclerosis [7,8]. The present study extends the same approach to breast cancer.

We therefore hypothesized that the expression levels of eight proliferation-, ferroptosis-, and immune-related targets (STAT3, GPX4, mTOR, PTEN, SLC7A11, miR-21, miR-210, miR-214) would differ between the peripheral blood mononuclear cells of breast cancer patients and those of healthy controls, and would show exploratory inter-target correlations. The primary objective was to compare the expression levels and directions of change of these eight targets between patients with breast cancer and healthy controls. The secondary objectives were to characterize inter-target correlations, to evaluate the exploratory discriminative performance of individual targets within the discovery cohort, to assess directional concordance in independent PBMC datasets, and to examine associations with clinicopathological features, particularly IHC-defined subtype.

## 2. Materials and Methods

### 2.1. Study Design and Ethical Approval

This cross-sectional, case-control study was approved by the Non-Interventional Clinical Research Ethics Committee of Atatürk University Faculty of Medicine (Decision no: B.30.2.ATA.0.01.00/395; date: 2 June 2026) and was conducted in accordance with the principles of the Declaration of Helsinki. The approval covers all 41 patients and 30 controls included in the study, including the six patients enrolled during the revision. Informed consent was obtained from all participants.

### 2.2. Study Group and Inclusion/Exclusion Criteria

The study included 41 female patients with histopathologically confirmed breast cancer and 30 healthy female controls with a similar age distribution. Healthy female controls were matched to the patient group by age (±5 years) and were screened through the same exclusion process as the patients. In both groups, the following were applied prospectively as exclusion criteria at enrolment: active infection; autoimmune or inflammatory disease; chronic inflammatory disease; malignancy other than the index breast cancer, including any personal history of malignancy; a hematological disorder that could affect blood sampling; recent major surgery; current use of systemic corticosteroids or other immunomodulatory agents; acute stress; obesity; diabetes mellitus; and current smoking. This strategy was adopted in order to limit the contribution of infectious, post-surgical, inflammatory, metabolic, endocrine and lifestyle-related states to the PBMC transcript profile, since all of these are known to modulate peripheral blood gene expression independently of tumor biology. Current smoking in particular was queried in every participant and current smokers were excluded from the patient group as well as from the control group, so that the two groups are balanced with respect to smoking exposure, which is a recognized modifier of leukocyte composition and gene expression. For the control group, it was additionally documented that no participant used hormonal contraception, had been recently vaccinated, reported a family history of breast cancer, or was pregnant, breastfeeding or within a recent pregnancy loss. Menopausal status was recorded in the control group; because equivalent verifiable information was not available for the patient group, this variable was not used in any analysis and is not reported. Obesity and current smoking were applied as categorical eligibility criteria rather than derived from tabulated values, and numerical body mass index and menstrual-cycle phase were not recorded in either group; this is stated as a limitation in Section 4. Clinicopathological data were extracted from the clinical and pathology records and are summarized in Section 3.2 and Supplementary Table S1. Documented variables comprised family history of breast cancer or known genetic predisposition, tumor laterality, BI-RADS category, focality, tumor size, axillary nodal involvement, disease status at presentation, estrogen and progesterone receptor and HER2 status, Ki-67 index, final surgical pathology, and neoadjuvant and surgical treatment. IHC-defined surrogate subtypes were assigned using prespecified criteria based on ER, PR, HER2 and Ki-67 status, and were derived from the documented immunohistochemical values rather than from the diagnostic label recorded in the clinical file. ER and PR positivity was defined as nuclear staining in at least 1% of tumor cells, whereas HER2 positivity was defined as an immunohistochemistry score of 3+ or amplification by in situ hybridization. Tumors negative for all three markers were classified as triple-negative. HER2-positive tumors were classified as HER2-enriched if estrogen-receptor negative, and as Luminal B (HER2-positive) if estrogen-receptor positive. Among HER2-negative, estrogen-receptor-positive tumors, those with a Ki-67 index below 20% and progesterone receptor expression of at least 20% were classified as Luminal A, and the rest as Luminal B (HER2-negative). Histological grade and a formally assigned TNM stage were not documented in the available records and are therefore not reported.

### 2.3. Blood Sample Collection and PBMC Isolation

Blood samples were collected from all patients after the diagnostic core-needle biopsy and histopathological confirmation of malignancy, but before definitive surgery and before any systemic therapy (chemotherapy, endocrine therapy, or radiotherapy). All patients were therefore treatment-naïve at the time of blood sampling. The 14 patients who subsequently received neoadjuvant systemic therapy were sampled before treatment initiation. The interval between the diagnostic biopsy and blood collection was not systematically recorded, so a transient, procedure-related contribution of the biopsy itself to PBMC gene expression cannot be completely excluded. Control samples were collected within the same time-of-day window as patient samples, under similarly standardized fasting conditions, to minimize circadian and metabolic contributions to PBMC gene expression. Peripheral venous blood samples were collected into EDTA tubes. Peripheral blood mononuclear cells (PBMCs) were isolated by density gradient centrifugation using Lymphoprep (den-sity 1.077 g/mL; Serumwerk Bernburg AG, Bernburg, Germany) in accordance with the manufacturer’s protocol. (Ficoll-Hypaque) in accordance with the manufacturer’s protocol. Following isolation, PBMCs were washed with phosphate-buffered saline (PBS) and total RNA was extracted from the freshly obtained cell pellet on the day of collection, without intermediate frozen storage of the pellet and without an additional stabilizing reagent. The extracted RNA was then stored at −80 °C until cDNA synthesis.

### 2.4. Total RNA Isolation

Total RNA was isolated from the freshly prepared PBMC pellets using the NucleoGene RNA Isolation Kit (NucleoGene, Istanbul, Türkiye) in accordance with the manufacturer’s instructions. To preserve the miRNA targets as well, isolation was performed according to a protocol covering the small RNA fraction. RNA concentration and purity were measured with a NanoDrop spectrophotometer (Thermo Fisher Scientific, Waltham, MA, USA); samples with an A260/A280 ratio in the 1.8–2.1 range were included in the study. RNA integrity was not assessed: no electrophoretic method or RNA integrity number (RIN) was used, and the A260/A280 ratio reported above is a measure of purity rather than of integrity. No DNase treatment was applied at any stage of the isolation procedure, so residual genomic DNA was not enzymatically removed. These limitations are stated in Section 4.

### 2.5. cDNA Synthesis

Complementary DNA (cDNA) was synthesized using the NucleoGene cDNA Synthesis Kit (NucleoGene, Istanbul, Türkiye) in accordance with the manufacturer’s protocol. For each sample, 500 ng to 1 µg of total RNA, contained in a volume of 16 µL, was combined with 4 µL of 5× cDNA synthesis reaction buffer to give a total reaction volume of 20 µL. The input was not identical across samples but varied within this range according to the RNA yield available for each sample. Reverse transcription was performed in a thermal cycler under the following conditions: 5 min at 25 °C, 30 min at 50 °C, and 5 min at 85 °C. For the miRNA targets (hsa-miR-21, hsa-miR-210, hsa-miR-214), the manufacturer’s miRNA-specific stem-loop/polyadenylation-based cDNA synthesis protocol was applied.

### 2.6. Quantitative Real-Time PCR (qRT-PCR)

Target expression was quantified using the NucleoGene Gene Expression Kit NucleoGene, (Istanbul, Türkiye) in accordance with the manufacturer’s protocol. Gene-specific primers were supplied as pre-validated components of this commercial kit in the form of a ready-to-use OligoMix. The complete panel is ordered from the manufacturer under a single catalog number (NGOT005), which covers the OligoMix as a whole rather than identifying individual assays, so the same catalog number applies to all ten assays used in this study. The primer sequences of the individual assays, for the mRNA targets as well as for the miRNA and SNORD61 assays, are proprietary to the manufacturer and were not disclosed to end users; they are therefore not reported here. The exact molar working concentrations of the supplied primer mixes were likewise not disclosed. Consequently no assay-level sequence information can be provided for any target, and this is stated as a limitation in Section 4. A mixture containing 5 µL of OligoMix, 10 µL of SYBR Green Master Mix, 4 µL of cDNA template, and 1 µL of RNase-free water was prepared, giving a total reaction volume of 20 µL. Each sample was prepared in duplicate. Amplification was performed on the Rotor-Gene Q Real-Time PCR System (Qiagen, Hilden, Germany). The cycling protocol consisted of an initial denaturation step at 95 °C for 15 min, followed by 40 cycles of 95 °C for 15 s and 60 °C for 60 s. Readings were recorded using the SYBR Green channel. Melting curve analysis was performed after amplification, ranging from 65 °C to 95 °C with 0.5 °C increments, holding for 5 s at each step. Amplification efficiency for each target was assessed by standard curve analysis using serial dilutions of pooled cDNA; efficiencies fell within the acceptable range of 95–105% (R^2^ ≥ 0.98). Melt-curve analysis gave a single, sharp dissociation peak for each target (typical Tm range 75–85 °C, varying by target), with no secondary peaks indicative of primer-dimer formation or nonspecific amplification. Each run included no-template controls (nuclease-free water substituted for cDNA); runs showing detectable amplification in the no-template control were reviewed and repeated when contamination or nonspecific amplification could not be excluded. All reactions were run in duplicate; technical duplicates with an inter-replicate Ct difference greater than 0.5 were repeated. Reverse transcription-negative (no-RT) controls were not included for any target. Melt-curve analysis yielded a single dissociation peak for every target and all no-template controls were negative, which supports the specificity of the amplification reactions. These observations do not, however, address residual genomic DNA in the samples themselves: a no-template control reports contamination of the reaction mix rather than of the template, and melt-curve analysis cannot reliably distinguish an amplicon of genomic origin. A contribution of genomic DNA therefore cannot be excluded, because neither DNase treatment nor no-RT controls were used. This is acknowledged as a methodological limitation in Section 4.

The targets examined were eight expression targets, comprising five mRNAs (STAT3, GPX4, mTOR, PTEN, SLC7A11) and three miRNAs (hsa-miR-21, hsa-miR-210, hsa-miR-214). The mRNA targets were normalized to GAPDH, and the miRNA targets to SNORD61 as the reference gene. Relative gene expression levels were calculated by the 2^−ΔΔCt^ method, using the mean ΔCt of the control group as the calibrator for each target.

### 2.7. Statistical Analysis

Statistical analyses were performed in Python 3.12.3 using SciPy 1.17.1, NumPy 2.4.4, pandas 3.0.2, statsmodels 0.15.0 and scikit-learn 1.8.0. The between-group comparability of the reference genes (GAPDH, SNORD61) was assessed by the independent-samples *t*-test and the coefficient of variation (CV); these procedures test whether reference gene Ct values differ between groups and do not constitute an algorithm-based reference-gene stability analysis (geNorm, NormFinder or BestKeeper), which was not performed. For each target, the normality of ΔCt values was tested by Shapiro–Wilk and the homogeneity of variance by the Levene test. In between-group comparisons, the independent-samples *t*-test was used for variables meeting the assumptions, and the Mann–Whitney U test for those that did not. The Benjamini–Hochberg false discovery rate (BH-FDR) correction was applied for multiple comparisons. To assess whether the group difference was independent of age, an analysis of covariance (ANCOVA) was fitted separately for each target, with the ΔCt value as the dependent variable, group (patient vs. control) as a fixed factor, and age as a continuous covariate; the age-adjusted group effect was subjected to the same Benjamini–Hochberg correction as the primary comparisons. Standardized effect sizes were calculated as Cohen’s d for targets compared by the independent-samples *t*-test and as the rank-biserial correlation for targets compared by the Mann–Whitney U test; post hoc power was not used as an effect-size measure. No a priori sample-size calculation was performed. In a post hoc sensitivity analysis, the available sample size (Patients *n* = 41, Controls *n* = 30) provides 80% power at two-sided α = 0.05 to detect effect sizes of approximately d ≥ 0.68, so that smaller true differences could have gone undetected. Relationships between targets were assessed by Spearman correlation; multiple-comparison correction was applied within predefined analysis families (primary expression: eight targets; correlation: 28 pairs). Exploratory discriminative performance was assessed by ROC analysis; the AUC and its 95% CI were determined by 2000-iteration bootstrap. No classification thresholds were derived, and sensitivity and specificity were therefore not estimated, since any cutoff selected and evaluated within a single cohort of this size would be optimistically biased. *p* < 0.05 was considered significant.

### 2.8. Analysis of Independent PBMC Datasets

The directional consistency of the mRNA targets was evaluated in two independent, publicly available peripheral blood mononuclear cell datasets obtained from the Gene Expression Omnibus (GEO).

GSE27562 was generated on the GPL570 platform (Affymetrix Human Genome U133 Plus 2.0 Array) and comprises 162 PBMC samples. The comparison used here was restricted to two groups: the malignant breast cancer samples (*n* = 57, the “Malignant” and “Pre-Surgery (aka Malignant)” annotations together) and the healthy female samples annotated “Normal” (*n* = 31). Benign breast disease (*n* = 37), ectopic malignancy (*n* = 22) and post-surgery (*n* = 15) samples were excluded, since they correspond to neither the case nor the control definition used here. This platform carries several probe sets per gene. We used every probe set annotated to a target gene in the GPL570 annotation and averaged their normalized values, giving one value per gene per sample. The rule was applied uniformly to every target and does not depend on the outcome. The probe sets used were: STAT3, 208991_at, 208992_s_at, 225289_at and 243213_at; MTOR, 202288_at and 215381_at; PTEN, 204053_x_at, 204054_at, 211711_s_at, 217492_s_at, 225363_at, 228006_at, 233254_x_at and 242622_x_at; SLC7A11, 207528_s_at, 209921_at and 217678_at. One PTEN probe set (217492_s_at) is annotated jointly to PTEN and to the PTEN pseudogene PTENP1; excluding it from the average did not change the direction of the PTEN result.

GSE47862 was generated on the GPL17279 platform and comprises 321 PBMC samples from the Utah (*n* = 248) and Ontario (*n* = 73) cohorts. The data are deposited as gene-level summarized values indexed by Entrez Gene identifier, so no probe-selection step was required; the values for STAT3 (Entrez 6774), MTOR (2475), PTEN (5728) and SLC7A11 (23657) were extracted directly. It should be noted that this dataset was not assembled as a diagnosed-patient versus healthy-control series. Participants were enrolled on the basis of breast cancer risk: 226 of the 321 women had a family history of breast cancer and 93 carried a BRCA mutation. The comparison available within the dataset is therefore between women who developed breast cancer (*n* = 158) and women who did not (*n* = 163), which addresses a different question from the case-control design of the present study. This difference in cohort construction should be taken into account when interpreting the directional discrepancies reported in Section 3.6, particularly because the control group of the present study explicitly excluded women with a family history of breast cancer, whereas familial risk was the basis of recruitment in GSE47862.

For both datasets, normalized expression values were used exactly as provided by the original submitters; no additional batch correction, renormalization or log transformation was applied, and no samples were excluded on the basis of missing values. Between-group comparisons within each dataset were performed with the Mann–Whitney U test. Because this analysis was an exploratory directional comparison across two independent datasets rather than a discovery analysis, *p*-values are reported as nominal and uncorrected for multiple testing, and should be interpreted accordingly. GPX4 is represented on both external platforms (GPL570 probe set 201106_at; Entrez Gene 2879) and is is reported in Section 3.6 together with the four targets that differed significantly in the present cohort, so that all five mRNA targets are shown. Because GPX4 did not differ significantly between patients and controls in the present cohort, its row is annotated accordingly and no directional inference is drawn from it.

For the miRNA targets, because no consistent representation was available in current PBMC datasets, directional consistency was instead informally compared with published findings from independent peripheral blood cohorts; this literature-based comparison does not constitute formal external validation.

## 3. Results

### 3.1. Study Population and Between-Group Comparability of the Reference Genes

The study included 41 patients with histopathologically confirmed breast cancer and 30 healthy controls. The groups were similar in age (Patients 37.7 ± 5.6 years; Controls 35.4 ± 5.6 years; *p* = 0.083). The reference genes GAPDH (*p* = 0.447, CV = 3.68%) and SNORD61 (*p* = 0.530, CV = 3.95%) showed comparable Ct values between the two groups, with low dispersion within each group (Table 1). These comparisons support the between-group comparability of the two reference transcripts under the present sampling conditions; they do not constitute formal reference-gene stability testing, and algorithm-based stability ranking (for example geNorm, NormFinder or BestKeeper) was not performed.

### 3.2. Clinicopathological Characteristics of the Patients

The clinicopathological characteristics of the patient group are summarized in Table 2. In the 38 cases with documented tumor size, the mean tumor size was 22.2 ± 14.0 mm, and most were 20 mm or smaller (24 of the 38 tumors with documented size, 63.2%; 24/41, 58.5% of the whole patient group). Thirty-two patients (78.0%) were node-negative, and seven (17.1%) had metastatic disease at presentation. In the IHC-defined surrogate subtype distribution, triple-negative was the most common subtype (*n* = 13, 31.7%), followed by Luminal B HER2-negative (*n* = 11, 26.8%), Luminal B HER2-positive (*n* = 11, 26.8%), and Luminal A (*n* = 6, 14.6%) tumors; no tumor met the criteria for the HER2-enriched (non-luminal) category. All patients were treatment-naïve at the time of blood sampling, and the 14 patients (34.1%) who received neoadjuvant systemic therapy did so after the sample had been obtained. Histological grade and a formally assigned TNM stage were not documented in the available clinical records and are therefore not reported. Additional characteristics, including laterality, BI-RADS category, family history, final surgical pathology and type of definitive surgery, are given in Supplementary Table S1.

### 3.3. Target Expression Levels

Seven of the eight targets examined differed significantly between the patient and control groups after BH-FDR correction (Table 3, Figure 1). STAT3, mTOR, SLC7A11, miR-21 and miR-210 were upregulated; PTEN and miR-214 were downregulated. The ferroptosis regulator GPX4 did not differ significantly between the groups (padj = 0.146). Standardized effect sizes for the significant targets ranged from moderate to large (Cohen’s d 0.59–1.33 in absolute value, and a rank-biserial correlation of 0.52 for mTOR), and are reported per target in Table 3 alongside the BH-FDR-corrected *p*-values and the log_2_FC and median fold-change values.

The possible confounding effect of age was assessed by ANCOVA. For each target, the ΔCt value was modeled as the dependent variable with group (patient vs. control) as a fixed factor and age as a continuous covariate. The age-adjusted group effect remained significant for all seven targets that were significant in the unadjusted analysis (BH-adjusted *p*: STAT3 0.000004; mTOR 0.000579; PTEN 0.000318; SLC7A11 0.002453; hsa-miR-21 0.046904; hsa-miR-210 0.000579; hsa-miR-214 0.002445), and GPX4 remained non-significant (BH-adjusted *p* = 0.147670). Age itself did not reach significance for any target after correction. The group difference in expression is therefore not accounted for by the age difference between the groups, although with the present sample size residual confounding cannot be excluded.

### 3.4. Exploratory Discriminative Performance

The exploratory discriminative performance of each target was assessed by single-marker ROC analysis (Table 4, Figure 2). These ROC estimates were derived and evaluated within the same discovery cohort and have not been tested externally. Cutoffs and their corresponding sensitivity and specificity are not reported, because any threshold derived in a cohort of this size and evaluated in the same cohort would be optimistically biased and would not be transferable to an independent population. The 2000-iteration bootstrap confidence intervals quantify sampling variability within the discovery cohort and should not be interpreted as independent validation. These findings should therefore be interpreted as preliminary and exploratory rather than as validated diagnostic utility. No multivariable or combined-marker classifier was constructed in this study; each AUC reflects a single target evaluated in isolation. These single-target estimates do not provide evidence of panel-level discriminative performance. Within these limits, the largest single-marker AUCs were observed for STAT3 (0.822), PTEN (0.779), hsa-miR-210 (0.763), mTOR (0.761), and SLC7A11 (0.742). The AUC for STAT3 was based on transcript abundance; activation of STAT3 signaling, which depends on tyrosine phosphorylation, was not assessed.

### 3.5. Inter-Target Correlations

In the patient group, relationships between the targets were examined by Spearman correlation; five correlations remained significant after BH-FDR correction (Table 5). The strongest were STAT3–GPX4 (ρ = +0.54) and hsa-miR-21–hsa-miR-210 (ρ = +0.54), followed by hsa-miR-21–hsa-miR-214 (ρ = +0.51), hsa-miR-210–hsa-miR-214 (ρ = +0.48) and GPX4–mTOR (ρ = +0.47). All three pairwise combinations of the measured miRNAs were positively correlated. These are transcript-level associations among co-measured targets in unfractionated cells and are reported as descriptive observations only.

### 3.6. Directional Consistency with Independent Datasets

The directional consistency of the five mRNA targets was examined in two independent PBMC datasets: GSE27562 (malignant *n* = 57, normal *n* = 31) and GSE47862 (cancer *n* = 158, control *n* = 163) (Table 6). SLC7A11 was upregulated in both external datasets and reached nominal significance in each (uncorrected *p* < 0.05; GSE27562, *p* = 0.013; GSE47862, *p* = 0.008). STAT3 was directionally consistent in both external datasets but did not reach statistical significance in either (GSE27562, *p* = 0.412; GSE47862, *p* = 0.283). mTOR was directionally consistent in only one of the two datasets. PTEN showed the opposite direction of change in both external datasets, although this discordance did not reach nominal significance in either (GSE27562, *p* = 0.073; GSE47862, *p* = 0.159). GPX4, which did not differ significantly between patients and controls in the present cohort, is shown in Table 6 for completeness and was directionally discordant in both external datasets without reaching nominal significance in either (*p* = 0.757 and *p* = 0.133). Overall, only SLC7A11 showed both directional and nominal statistical consistency across the two external datasets; the remaining targets showed limited or no consistency. The two external datasets had substantially different study designs, which should be considered when interpreting discrepancies in directional concordance. GSE27562 compares diagnosed malignant cases with healthy women. GSE47862 compares women who developed breast cancer with women who did not, within a cohort enriched for familial breast cancer risk and BRCA mutation carriers (Section 2.8). Directional concordance with the present case-control study may therefore be more interpretable for GSE27562 than for GSE47862.

For the miRNA targets, directional consistency was informally compared with published peripheral blood findings; this literature-based comparison does not constitute formal external validation. The upregulated hsa-miR-21 is consistent with a study reporting an increase in PBMCs in breast cancer [9]; the positive correlation between hsa-miR-21 and hsa-miR-210 (ρ = +0.54) is consistent with an independent report of a strong positive correlation between these two miRNAs (r = 0.70 in tumor tissue and r = 0.75 in adjacent normal tissue) [10]; that comparison was made in tissue rather than in peripheral blood, so it indicates a shared regulatory pattern rather than a compartment-matched replication. Direct PBMC data are limited for hsa-miR-214.

### 3.7. Relationship of Target Expression with Clinicopathological Parameters

In the patient group, the relationship between target expression levels and clinicopathological parameters was evaluated. No statistically significant relationship was found, after multiple-comparison correction, between target expression and receptor status, lymph node involvement, the Ki-67 index, or IHC-defined subtype (all BH-FDR *p* > 0.05). In a post hoc exploratory TNBC (*n* = 13) vs. non-TNBC (Luminal A and Luminal B, HER2-negative and HER2-positive, combined; *n* = 28) comparison, PTEN expression was lower in TNBC cases (relative expression 0.251) than in non-TNBC cases (0.444), but the difference did not survive correction across the eight targets (Mann–Whitney U, *p* = 0.024; BH-FDR *p* = 0.193).

The behavior of this comparison as the cohort grew is worth recording. In the first 35 patients, the same test was significant after correction (relative expression 0.423; *p* = 0.004, BH-FDR *p* = 0.034). After six further patients were enrolled, two of whom had triple-negative disease, the effect size approximately halved and the corrected *p*-value rose well above the significance threshold. This instability is, in itself, informative: it shows how fragile subgroup-level associations are at these subgroup sizes. Accordingly, no clinicopathological association is claimed for PTEN or for any other target in this study, and any such relationship would require testing in a prespecified, adequately powered and compartment-matched cohort.

## 4. Discussion

The expression profile of eight proliferation-, ferroptosis- and immune-related targets was evaluated in PBMCs from patients with breast cancer. Seven of the eight targets (STAT3, mTOR, PTEN, SLC7A11, miR-21, miR-210, miR-214) differed significantly between patient and control groups. The findings support the possibility that PBMC expression patterns capture aspects of the systemic host response associated with breast cancer. Tumor–host interactions are not confined to the local microenvironment: systemic immune and metabolic reprogramming leaves detectable traces in peripheral blood cells [2].

Among the proliferation- and survival-related targets, the upregulation of miR-21 and miR-210 is consistent with several earlier reports. Previous studies have linked miR-21-mediated PTEN suppression to sustained signaling through the PI3K/AKT/mTOR survival pathway [3]. Here, the concordant expression directions of miR-21, PTEN and mTOR resemble that pattern; however, the present data were obtained from bulk PBMCs at the transcript level and do not establish a functional regulatory cascade in this compartment. The positive correlation between miR-21 and miR-210 observed in the present study (ρ = +0.51) is consistent with an independent report of a strong positive correlation (r≈0.70–0.75) between these two miRNAs in breast cancer [10].

Turning to the ferroptosis-associated targets, the upregulation of SLC7A11 was among the most consistent expression findings. In the literature, SLC7A11 is reported to enable cystine uptake, feed glutathione (GSH) biosynthesis, support GPX4-mediated lipid peroxide detoxification, and thereby suppress ferroptosis [5]; our data show only that SLC7A11 transcript levels were elevated in patients, not that ferroptosis resistance was functionally demonstrated in this cohort. A notable exploratory observation is that GPX4, which showed no significant difference at the group level, exhibited significant positive correlations with mTOR (ρ = +0.46) and STAT3 (ρ = +0.51). This pattern is consistent with a previously described mechanism in the literature, whereby SLC7A11-mediated cystine uptake promotes GPX4 protein synthesis through the Rag–mTORC1–4EBP signaling axis [11]; our correlational data are compatible with, but do not directly test, this mechanism. Post-transcriptional regulation of GPX4 has been proposed in the literature as one possible reason why transcript-level measurement may not track GPX4 activity; this was not tested here and is offered only as a candidate explanation for its weak single-marker performance in peripheral blood.

For the targets related to immune-metabolic regulation, STAT3 was upregulated and reached a single-marker AUC of 0.822 in this cohort; as this estimate was not externally validated and reflects only one discovery-cohort analysis, it is reported here as a preliminary observation rather than a validated diagnostic result. STAT3 has been closely associated with the expansion of myeloid-derived suppressor cells, immune evasion, and chemotherapy resistance in the tumor microenvironment [6]. Prior experimental work has shown that STAT3 can suppress ferroptosis by transcriptionally increasing the expression of SLC7A11 and GPX4 [12]; this reported mechanism offers a plausible biological explanation for the STAT3–GPX4 and STAT3–mTOR correlations we observed, although our bulk mRNA data cannot establish this mechanism directly.

Regarding the tumor-suppressor-associated targets, downregulation of PTEN and miR-214 was observed. The role of miR-214 in breast cancer is contradictory in the literature: some studies define it as an oncogene targeting p53 [13], whereas others attribute a tumor-suppressor function [14]. The observed decrease in miR-214 expression is consistent with reports supporting a tumor-suppressive role for this miRNA. A further exploratory observation concerns the relationship of peripheral blood PTEN expression with IHC-defined subtype. PBMC PTEN expression was numerically lower in patients with TNBC than in patients with non-TNBC disease, a pattern that would be biologically compatible with reports of frequent PTEN loss in TNBC tumor tissue; however, the difference did not survive multiple-testing correction in the present cohort, and tumor-tissue PTEN loss should in any case not be equated with reduced PTEN transcript abundance in PBMCs. PTEN showed the opposite direction of change in both external datasets compared with the present cohort. Possible explanations include compartment differences (PTEN expression in PBMCs may be determined by systemic immune cell composition), platform differences, and cohort composition differences. Therefore, our PTEN findings should be interpreted as cohort-specific.

Collectively, the observed expression changes can be grouped descriptively into proliferation-related alterations involving miR-21, PTEN and mTOR; ferroptosis-related alterations involving SLC7A11 together with the correlations of GPX4 with mTOR and STAT3; and increased STAT3 expression related to immune-metabolic regulation. These groupings rest on previously reported biological functions and do not demonstrate coordinated regulation or functional pathway activation in PBMCs. Within the clinicopathological analyses, no association survived multiple-testing correction. The nominal difference in peripheral blood PTEN expression between TNBC and non-TNBC cases is reported for completeness and is not put forward as a finding of this study.

To keep the different levels of evidence in this study visually distinct, the findings are summarized in Figure 3. The figure separates what was measured in PBMCs from what is only inferred and was not assayed, and shows the external directional status of each mRNA target alongside the interpretive limits that apply to the whole dataset.

These findings are best read against the earlier peripheral blood expression literature, which is summarized in Table 7. Two features of that literature are relevant here. First, the studies that achieved the most convincing performance did so with genome-wide platforms and multigene classifiers, and only one of them validated its model in a truly independent cohort. Second, the cohorts differ substantially in what the comparison group represents, ranging from population controls to women referred for diagnostic mammography and to women with familial risk who had not developed cancer, so reported accuracies are not directly comparable across studies. The present study occupies a different position: it uses a small, targeted qRT-PCR panel rather than a genome-wide platform, does not construct a classifier, and reports directional comparison with public datasets rather than external validation. Its contribution is therefore hypothesis-generating rather than diagnostic, and its cohort size is at the lower end of this literature.

The study has some limitations. First, the study has a single-center, single-cohort (41 patients, 30 controls), cross-sectional, and exploratory design without an independent validation cohort. Second, all measurements were performed at the RNA/transcript level. No protein abundance, phosphorylation or functional activity was assessed, including phospho-STAT3 and GPX4 protein activity. Pathway activation and ferroptosis resistance were therefore not demonstrated. Both remain inferences drawn from transcript-level associations reported in the literature. Relatedly, transcripts were measured in bulk, unfractionated PBMCs. Cell-composition data, such as monocyte, lymphocyte and NK-cell proportions, were not obtained. The reported differences may therefore reflect a shift in circulating immune-cell composition rather than altered regulation within a fixed cell population, or a combination of both. Future studies using cell-sorted or single-cell approaches, together with protein-level assays, are needed to distinguish between these possibilities.

Third, the subtype subgroups are small (TNBC *n* = 13; Luminal A *n* = 6). The nominal PTEN-subtype difference rests on a within-cohort comparison, did not survive multiple-testing correction, and could not be evaluated in the external datasets, because neither dataset included IHC-defined subtype information. Tumor-tissue datasets were not used because they would not provide compartment-matched validation of a PBMC-based finding. Fourth, because the study had a cross-sectional design, causality and prognostic value could not be assessed. Fifth, discriminative performance was assessed only at the single-marker level and only as an exploratory analysis; no multivariable or combined-panel classifier was constructed, and ROC performance was not validated in an independent cohort, so the reported AUCs should be regarded as preliminary, discovery-cohort estimates rather than as validated diagnostic tools. Future studies should include larger, compartment-matched cohorts, prespecified sample-size calculations, combined transcript and protein measurements, longitudinal follow-up, and independently validated multivariable classifiers. Sixth, histological grade and a formally assigned TNM stage were unavailable in the clinical records accessible for this cohort. We therefore reported the documented individual components of disease burden, including the largest tumor diameter, axillary nodal involvement, and metastatic status at presentation, rather than reconstructing a composite stage retrospectively. Consequently, the relationships between the PBMC expression profile, tumor differentiation, and formally staged disease extent could not be evaluated. To assess whether disease burden nonetheless confounded the expression differences, we compared the eight targets within the patient group according to the documented burden indicators, that is, largest tumor diameter above versus at or below 20 mm and node-positive versus node-negative status (Supplementary Table S3). No comparison was significant after BH-FDR correction in either stratification, which argues against a strong burden-driven contribution, although both subgroup comparisons are underpowered and cannot exclude a modest effect. Because seven patients had documented metastatic disease at presentation, the case-control comparison was repeated after excluding them (Supplementary Table S2); six of the seven originally significant targets retained significance, whereas hsa-miR-21 did not. Seventh, two aspects of the qRT-PCR workflow were not covered. DNase treatment was not applied and no-RT controls were not run, so a contribution from residual genomic DNA cannot be excluded. The melt-curve and no-template control results support amplification specificity, but neither addresses genomic DNA present in the template, and this limitation is compounded by the fact that the proprietary assay sequences do not allow exon-exon junction spanning to be verified. In addition, the two reference genes were selected a priori and were evaluated only for between-group comparability of Ct values, without algorithm-based stability ranking, so the normalization strategy was not formally validated for this sample type. A further reporting constraint follows from the assay format: the OligoMix panel is supplied under a single catalog number covering all assays, and the primer sequences of the individual assays are proprietary to the manufacturer, so no target-level sequence identifier can be reported. As a result, the amplicons cannot be verified independently, it cannot be confirmed whether the assays span exon-exon junctions, and the transcript variants covered by each assay cannot be determined from the information available to us. Eighth, although obesity, diabetes mellitus, chronic inflammatory disease, recent major surgery, current corticosteroid or immunomodulatory treatment and current smoking were applied as exclusion criteria in both groups, these were categorical eligibility decisions and the underlying quantitative variables were not tabulated; numerical body mass index and menstrual-cycle phase were not recorded, and the operational definition of acute stress was clinical rather than instrument-based. The intervals implied by “recent” for vaccination, major surgery and pregnancy loss were applied at the clinician’s discretion and were not prespecified as fixed time windows, and subclinical inflammation was not assessed objectively, since C-reactive protein or comparable inflammatory markers were not measured in either group. Residual confounding by graded or subclinical variation in these factors therefore cannot be fully excluded, although age matching within ±5 years, the uniform exclusion process applied to both groups, and standardized time-of-day and fasting conditions at sampling substantially reduce it. Ninth, the enrolled cohort was comparatively young (patients 37.7 ± 5.6 years; controls 35.4 ± 5.6 years), which may partly reflect the exclusion criteria described in Section 2.2, since chronic inflammatory disease, obesity, diabetes mellitus, corticosteroid or immunomodulatory treatment and current smoking all become more prevalent with advancing age. We did not maintain a screening log recording the age distribution of candidates who were assessed but not enrolled, so we cannot quantify this effect and present it as a plausible contributor rather than an established mechanism. This increases internal comparability between the patient and control groups and reduces within-study confounding, but it limits the generalizability of the findings to postmenopausal and older breast cancer populations. Several age-related mechanisms could plausibly alter the targets examined here independently of tumor biology: immunosenescence and chronic low-grade inflammation, described as inflammaging, may raise baseline NF-κB and IL-6/STAT3 signaling in circulating leukocytes [20,21]; the loss of estrogen receptor signaling after menopause, which otherwise restrains NF-κB at several regulatory steps and modulates PI3K/AKT and related signaling in lymphocytes and monocytes, may shift the basal expression of mTOR- and PTEN-related targets [22,23]; and the subclinical metabolic dysfunction that accumulates with age, including insulin resistance and dyslipidemia, may affect the ferroptosis-related targets examined here, particularly SLC7A11, since iron handling and lipid peroxidation are altered in type 2 diabetes and related metabolic states [24]. These are mechanistic possibilities rather than parameters measured in the present study, and they indicate that the present findings require confirmation in age-stratified and postmenopausal cohorts.

## 5. Conclusions

Seven of the eight measured targets showed differential expression in bulk PBMCs from patients with breast cancer, and five significant pairwise correlations were identified among them. In the clinicopathological analyses, no association survived multiple-testing correction; the nominally lower PBMC PTEN expression in TNBC cases was not robust to enlargement of the cohort and is not advanced as a finding. Only SLC7A11 showed both directional concordance and nominal statistical significance in both external datasets; evidence for the remaining targets was limited or discordant. Because the study assessed transcript levels in unfractionated PBMCs without protein-level, cell-composition or functional measurements, the findings should be interpreted as hypothesis-generating expression patterns rather than as evidence of pathway activation or ferroptosis resistance. Validation in larger, independent, compartment-matched cohorts is warranted.

## Figures and Tables

**Figure 1:**
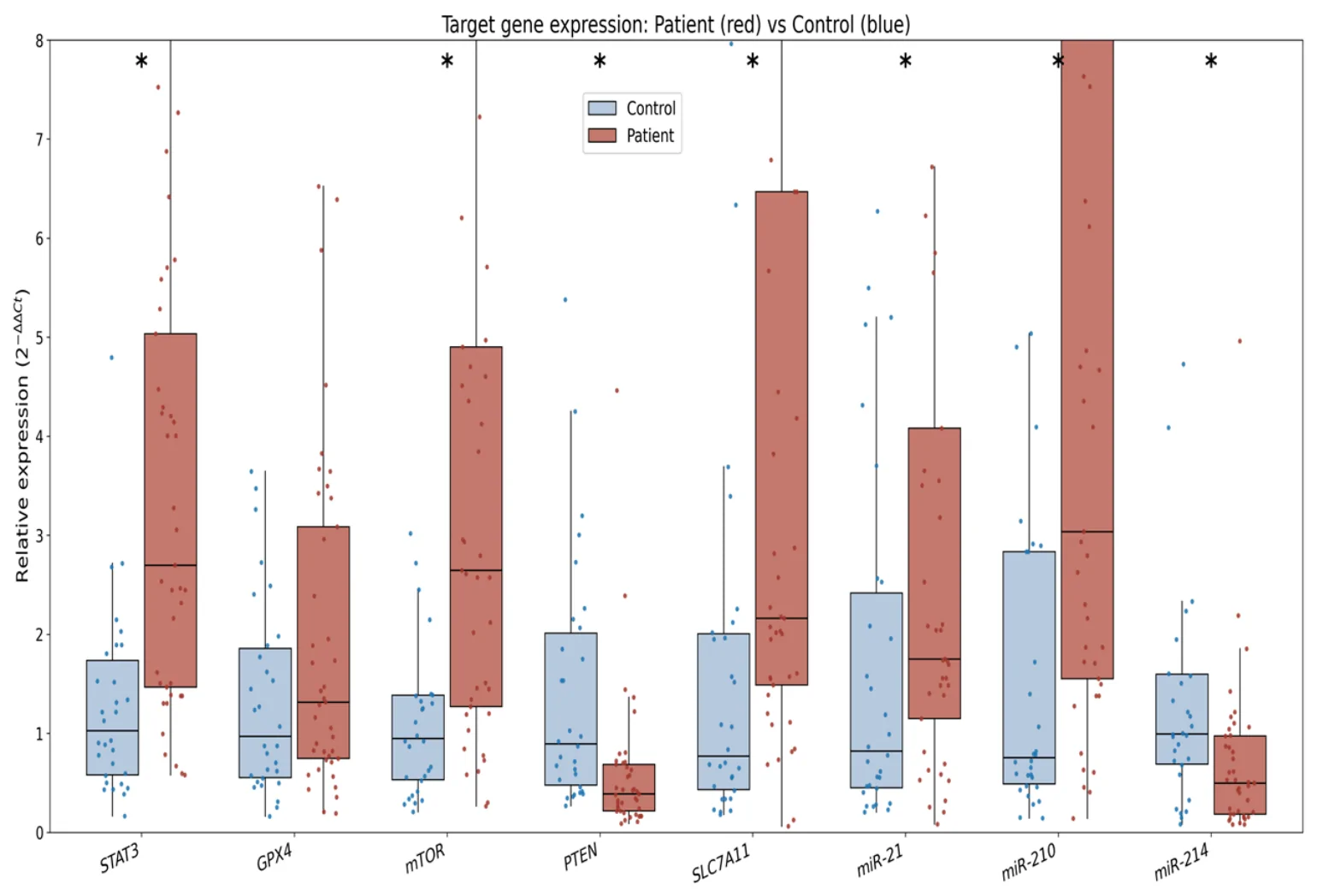
Relative expression distributions of the expression targets by group (box plot). Asterisk (*) indicates targets significant after BH-FDR correction; GPX4 is not significant.

**Figure 2:**
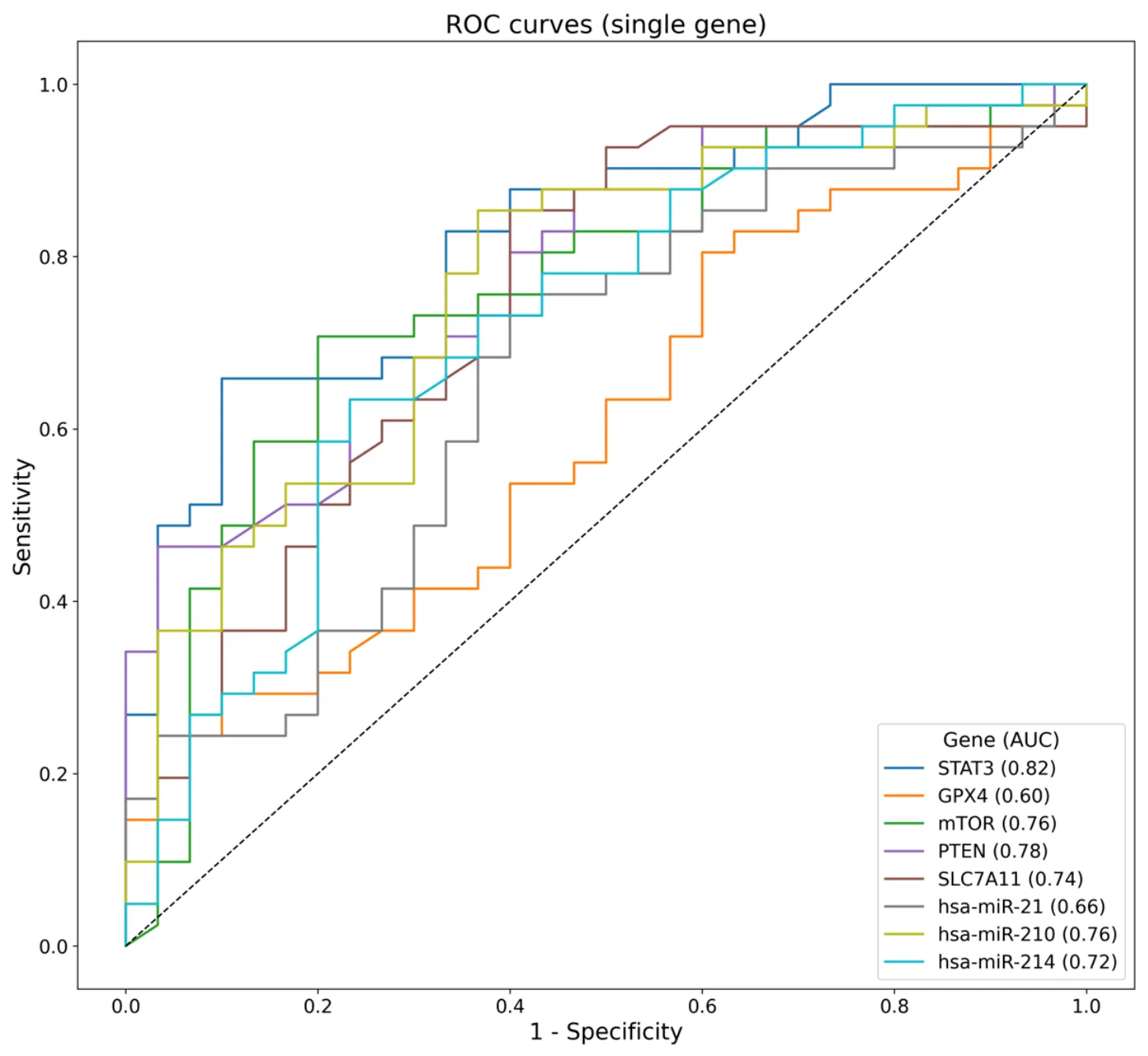
Single-marker ROC curves and AUC values for the eight expression targets.

**Figure 3:**
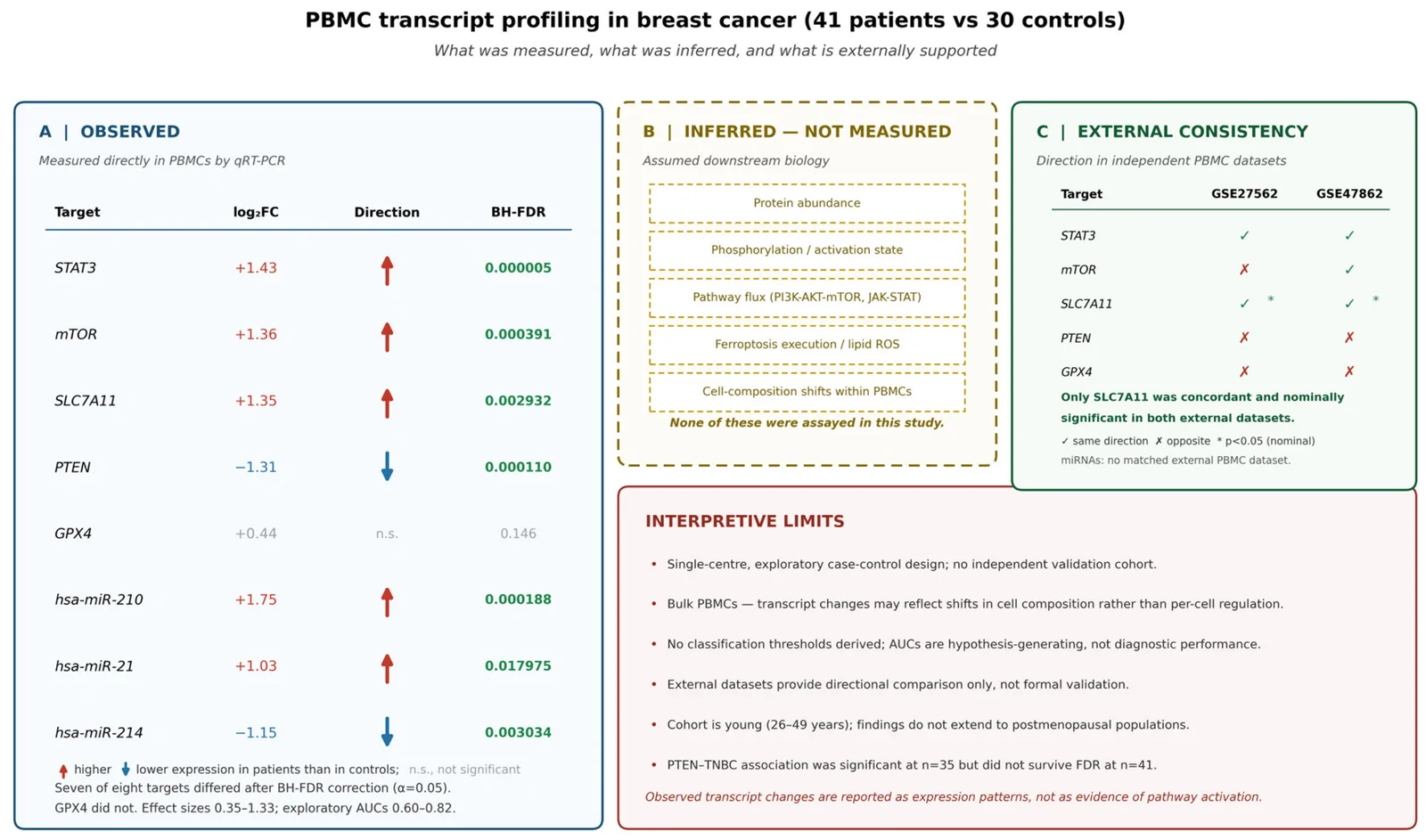
Summary of the study findings, separating observed PBMC transcript changes from inferred biology and from external directional support. (**A**) Observed: direction, log_2_FC and BH-FDR status of the eight targets measured by qRT-PCR in PBMCs (41 patients vs. 30 controls). (**B**) Inferred but not measured: protein abundance, phosphorylation and activation state, pathway flux, ferroptosis execution and PBMC cell-composition shifts; none of these were assayed in this study. (**C**) External consistency: direction of each mRNA target in GSE27562 and GSE47862, with an asterisk marking nominal significance (*p* < 0.05, uncorrected); only SLC7A11 was concordant and nominally significant in both. The lower panel lists the interpretive limits that apply to all three tiers. No miRNA target could be assessed externally because no matched public PBMC dataset was available.

**Table 1:** Demographic characteristics and between-group comparison of reference gene Ct values.

Variable	Control (*n* = 30)	Patient (*n* = 41)	*p*
Age (years, mean ± SD)	35.4 ± 5.6	37.7 ± 5.6	0.083
GAPDH (Ct)	23.59 ± 0.96	23.75 ± 0.87	0.447
SNORD61 (Ct)	24.26 ± 0.95	24.41 ± 0.97	0.530

Mann–Whitney U for age; independent-samples *t*-test for reference gene Ct.

**Table 2:** Clinicopathological characteristics of the patient group (*n* = 41).

Characteristic	*n*	% (of 41)
Demographic characteristics		
Age (years, mean ± SD)	37.7 ± 5.6	—
Tumor burden		
Unifocal	14	34.1
Multifocal	15	36.6
Multicentric	11	26.8
Focality not stated	1	2.4
Tumor size (mm, mean ± SD; *n* = 38) a	22.2 ± 14.0	—
Largest tumor diameter ≤20 mm a	24	58.5
Largest tumor diameter 21–50 mm a	12	29.3
Largest tumor diameter >50 mm a	2	4.9
Tumor size not documented	3	7.3
Node-negative b	32	78.0
1–3 positive axillary nodes b	6	14.6
≥4 positive axillary nodes b	3	7.3
Non-metastatic at presentation	33	80.5
Metastatic at presentation c	7	17.1
Disease status not clearly documented	1	2.4
Receptor status and IHC-defined surrogate subtype		
Estrogen receptor positive (≥1%)	28	68.3
Estrogen receptor negative	13	31.7
Progesterone receptor positive (≥1%)	27	65.9
Progesterone receptor negative	14	34.1
HER2 positive	11	26.8
HER2 negative	30	73.2
Ki-67 index (%, median [IQR]) d	22.5 [10.0–32.5]	—
Luminal A e	6	14.6
Luminal B, HER2-negative e	11	26.8
Luminal B, HER2-positive e	11	26.8
Triple-negative e	13	31.7
Treatment status and timing of blood sampling		
Treatment-naïve at the time of blood sampling	41	100
Neoadjuvant systemic therapy, administered after blood sampling	14	34.1
No neoadjuvant systemic therapy	27	65.9
Variables not available in the clinical records		
Histological grade f	Not available	—
Formal TNM stage f	Not available	—

Percentages are calculated over the whole patient group (*n* = 41) and may not total 100 owing to rounding. a—Tumor size was documented for 38 of 41 patients and is reported as the largest recorded diameter, measured at diagnosis and before any treatment; the size categories are descriptive groupings and do not constitute a formally assigned TNM T category. b—Number of metastatic axillary lymph nodes documented at sentinel lymph node biopsy; in one patient an additional axillary dissection was recorded without a documented node count, so this patient may belong to a higher category. c—Metastatic disease at presentation as recorded in the clinical file; patients with axillary nodal involvement but no distant spread are counted as non-metastatic. Nodal status and metastatic status are reported independently of one another: four of the seven patients with documented metastatic disease at presentation were node-negative, and the remaining three had one or more metastatic axillary nodes. d—Ki-67 values recorded as ranges were summarized at the midpoint of the range. e—Surrogate subtypes were assigned from the documented immunohistochemical values using the prespecified criteria given in Section 2.2, not from the diagnostic label recorded in the clinical file; no tumor met the criteria for the HER2-enriched (non-luminal) category because every HER2-positive tumor in this cohort was also estrogen-receptor positive. f—Histological grade and formal TNM stage were not documented in the available clinical records for any patient (see Limitations). Additional clinicopathological characteristics are given in Supplementary Table S1.

**Table 3:** Target expression differences (Patient vs. Control).

Gene	RQ (Patient)	log_2_FC	Median FC	Direction	*p* (FDR)	Effect Size a
STAT3	3.48	+1.43	2.70	↑	0.000005	+1.33
GPX4	1.96	+0.44	1.32	↑	0.146	+0.35
mTOR	4.13	+1.36	2.65	↑	0.000391	+0.52 b
PTEN	0.60	−1.31	0.39	↓	0.000110	−1.08
SLC7A11	4.86	+1.35	2.16	↑	0.002932	+0.78
hsa-miR-21	4.30	+1.03	1.75	↑	0.017975	+0.59
hsa-miR-210	7.17	+1.75	3.04	↑	0.000188	+1.02
hsa-miR-214	0.71	−1.15	0.50	↓	0.003034	−0.76

RQ: 2^−ΔΔCt^. mRNA targets normalized to GAPDH, miRNA targets to SNORD61. BH-FDR-corrected *p*-values are reported to six decimal places where they fall below 0.001. a—Effect size expressed so that positive values indicate higher expression in patients: Cohen’s d computed on ΔCt values for targets compared by the independent-samples *t*-test. b—For mTOR, which was compared by the Mann–Whitney U test, the rank-biserial correlation is given instead of Cohen’s d. ↑ indicates upregulation; ↓ indicates downregulation.

**Table 4:** Exploratory single-target discriminative performance (ROC-AUC).

Gene	AUC	95% CI
STAT3	0.822	0.721–0.907
PTEN	0.779	0.663–0.879
hsa-miR-210	0.763	0.638–0.875
mTOR	0.761	0.635–0.872
SLC7A11	0.742	0.624–0.850
hsa-miR-214	0.718	0.590–0.837
hsa-miR-21	0.663	0.526–0.790
GPX4	0.600	0.464–0.729

AUC: area under the curve; 95% CI by 2000-iteration bootstrap. The bootstrap confidence interval reflects sampling variability within this cohort and does not constitute independent validation. No classification thresholds were derived, and sensitivity and specificity are therefore not reported; the AUC values are presented as exploratory, hypothesis-generating estimates rather than as validated diagnostic utility.

**Table 5:** Significant inter-gene correlations after BH-FDR (Patient group).

Target Pair	Spearman ρ	*p*	*p* (FDR)
STAT3 ↔ GPX4	+0.54	<0.001	0.004
hsa-miR-21 ↔ hsa-miR-210	+0.54	<0.001	0.004
hsa-miR-21 ↔ hsa-miR-214	+0.51	0.001	0.006
hsa-miR-210 ↔ hsa-miR-214	+0.48	0.001	0.010
GPX4 ↔ mTOR	+0.47	0.002	0.012

ρ: Spearman correlation coefficient. BH-FDR applied for the 28 possible pairs; only pairs significant after correction are listed.

**Table 6:** Directional consistency of the mRNA targets in independent PBMC datasets.

Gene	This Study	GSE27562	p_1_	GSE47862	p_2_	Concordance
STAT3	↑	↑	0.412	↑	0.283	2/2
mTOR	↑	↓	0.003	↑	0.192	1/2
PTEN	↓	↑	0.073	↑	0.159	0/2
SLC7A11	↑	↑	0.013	↑	0.008	2/2 *
GPX4	↑ (ns)	↓	0.757	↓	0.133	Not assessed

Direction: change in the case group relative to the control group within each dataset. GSE27562: malignant breast cancer (*n* = 57) vs. normal (*n* = 31). GSE47862: women who developed breast cancer (*n* = 158) vs. women who did not (*n* = 163), in a cohort enriched for breast cancer risk (see Section 2.8). *p*: Mann–Whitney U, uncorrected (nominal) values; no multiple-testing correction was applied across the two external datasets given their exploratory, directional-comparison purpose. GPX4 did not differ significantly between patients and controls in the present cohort (BH-adjusted *p* = 0.146); its row is shown for completeness and no directional inference is drawn from it. * SLC7A11 was directionally concordant and nominally significant (*p* < 0.05, uncorrected) in both external datasets (GSE27562, *p* = 0.013; GSE47862, *p* = 0.008). ↑ indicates upregulation; ↓ indicates downregulation.

**Table 7:** Summary of previous peripheral blood and PBMC expression studies in breast cancer.

Study	Cohort	Sample	Platform	Targets or Signature	Validation Approach	Key Limitation
Sharma et al., 2005 [15]	24 breast cancer/32 without disease	Peripheral blood cells	Macroarray (1368 genes)	37-gene predictor	Leave-one-out cross-validation within the same cohort	Small cohort; no independent validation set
Aarøe et al., 2010 [16]	67 breast cancer/54 non-malignant (plus 6 healthy)	Peripheral blood cells	High-density oligonucleotide microarray	Multigene PLSR predictor	Leave-one-out double cross-validation within the same cohort	Comparison group referred for diagnostic mammography rather than population controls
LaBreche et al., 2011 [17] (GSE27562)	57 malignant/31 normal (subset analyzed here)	PBMC	Genome-wide microarray	Latent-factor blood predictor	Internal cross-validation; cross-species model transfer	Predictor developed from a mouse model; no prospective clinical validation
Piccolo et al., 2015 [18] (GSE47862)	124 (Utah, training)/73 (Ontario, test)	PBMC	Genome-wide microarray	Support vector machine multigene biomarker	External validation in an independent cohort (AUC 0.76 and 0.73)	Cohort enriched for familial risk and BRCA1/2 carriers; not a general case-control comparison
Tudoran et al., 2014 [19]	30 ER-negative, PR-negative patients (HER2-positive vs. triple-negative)	Peripheral blood	Targeted qPCR array (84 genes)	15 differentially expressed genes	None; single-cohort analysis	No healthy control group; comparison restricted to two tumor subtypes
Present study	41 breast cancer/30 healthy controls	PBMC	Targeted qRT-PCR (8 targets)	7 of 8 targets differentially expressed	Directional comparison in two public PBMC datasets; no formal external validation	Small cohort; no classification thresholds derived; transcript level only

PBMC: peripheral blood mononuclear cells; PLSR: partial least squares regression; AUC: area under the curve. Cohort sizes are as reported in the original publications; for GSE27562 and GSE47862 the numbers correspond to the samples analyzed in the present study and in the original report, respectively.

## Data Availability

The datasets generated and analyzed during the current study are available from the corresponding author on reasonable request.

## Supplementary Materials

The following supporting information can be downloaded at: https://www.mdpi.com/article/10.3390/medicina62101831/s1, Table S1, Additional clinicopathological characteristics of the patient group (*n* = 41); Table S2, Case-control comparison of the eight targets before and after exclusion of the seven patients with documented metastatic disease at presentation; Table S3, Sensitivity analysis of target expression with respect to tumor burden within the patient group (*n* = 41).
