# Supplementary material for: Peripheral Blood Mononuclear Cell Expression Profile of Proliferation-, Ferroptosis-, and Immune-Related Targets in Breast Cancer
Source: Medicina (Kaunas). 2026 Sep 23;62(10):1831. doi: 10.3390/medicina62101831 (PMC13707144; doi:10.3390/medicina62101831)
Supplement: Supplementary file 1 [file medicina-62-01831-s001.zip › medicina-4504804-Supplementary Materials.pdf]

Supplementary Materials

The following supporting information is available: Table S1, additional clinicopathological characteristics of the patient group (n=41); Table S2, case-control comparison of the eight targets before and after exclusion of patients with documented metastatic disease at presentation; Table S3, sensitivity analysis of target expression with respect to tumor burden within the patient group.

Table S1. Additional clinicopathological characteristics of the patient group (n=41).

Characteristic	n	% (of 41)
**Tumor laterality**		
Left	20	48.8
Right	17	41.5
Bilateral	4	9.8
**BI-RADS category**		
BI-RADS 4a	5	12.2
BI-RADS 4 (subcategory not specified)	7	17.1
BI-RADS 4c	7	17.1
BI-RADS 5	13	31.7
BI-RADS 6	9	22.0
**Family history of breast cancer or known genetic predisposition**		
Present	15	36.6
Absent	26	63.4
**Additional descriptive statistics**		
Age (years, median [range])	38 [26–49]	—
Tumor size (mm, median [range]; n=38)	17.2 [6–70]	—
Ki-67 <20%	20	48.8
Ki-67 ≥20%	21	51.2
**Final surgical pathology**		
Invasive ductal carcinoma (NST), including microinvasive cases	20	48.8
Invasive carcinoma, not otherwise specified	7	17.1
Invasive papillary carcinoma	6	14.6

Characteristic	n	% (of 41)
Invasive tubular carcinoma	2	4.9
Mixed histology	2	4.9
Invasive lobular carcinoma	1	2.4
Ductal carcinoma in situ only	1	2.4
No residual invasive or in situ carcinoma after neoadjuvant therapy	2	4.9
**Definitive surgery (performed after blood sampling)**		
Simple mastectomy	24	58.5
Breast-conserving surgery	13	31.7
Modified radical mastectomy	3	7.3
Segmental mastectomy	1	2.4

Percentages are calculated over the whole patient group (n=41) and may not total 100 owing to rounding. NST, no special type.

Table S2. Case-control comparison of the eight targets before and after exclusion of the seven patients with documented metastatic disease at presentation.

Target	RQ (full cohort)	BH-adjusted p (full cohort, n=41)	RQ after exclusion	BH-adjusted p after exclusion (n=34)
STAT3	3.48	0.000005	3.18	0.000061
GPX4	1.96	0.146	1.71	0.297
mTOR	4.13	0.000391	4.23	0.000572
PTEN	0.60	0.000110	0.46	0.000018
SLC7A11	4.86	0.002932	4.37	0.015012
hsa-miR-21	4.30	0.017975	3.31	0.076935
hsa-miR-210	7.17	0.000188	7.54	0.000572
hsa-miR-214	0.71	0.003034	0.60	0.002311

RQ is the mean of the individual $2^{-\Delta\Delta Ct}$ values. Between-group comparisons and the choice of test follow Section 2.7: the independent-samples t-test was used for targets meeting the normality and variance assumptions and the Mann-Whitney U test otherwise, with Benjamini-Hochberg correction applied within each analysis. The full-cohort column reproduces Table 3. The 34 patients retained after exclusion comprise the 33 patients documented as non-metastatic at presentation and one patient whose disease status was not clearly documented; repeating the analysis with this patient also excluded (n=33) changed no conclusion for any target.

Table S3. Sensitivity analysis of target expression with respect to tumor burden within the patient group (n=41).

Target	p (tumor size >20 mm vs. ≤20 mm)	BH-adjusted p (tumor size)	p (node-positive vs. node-negative)	BH-adjusted p (nodal status)
STAT3	0.5755	0.8678	0.3446	0.3446
GPX4	0.7507	0.8678	0.1912	0.3233
mTOR	0.1463	0.8678	0.0751	0.2004
PTEN	0.7970	0.8678	0.2021	0.3233
SLC7A11	0.8678	0.8678	0.3288	0.3446
hsa-miR-21	0.5450	0.8678	0.0472	0.1888
hsa-miR-210	0.4226	0.8678	0.2772	0.3446
hsa-miR-214	0.3968	0.8678	0.0189	0.1515

Mann-Whitney U tests within the patient group; BH-FDR applied across the eight targets separately for each comparison. Tumor size was documented for 38 of 41 patients (>20 mm, n=14; ≤20 mm, n=24). Nodal status was derived from the documented number of metastatic sentinel nodes (node-positive n=9; node-negative n=32). No comparison remained significant after correction.
